# Therapeutic Opportunities of GPBAR1 in Cholestatic Diseases

**DOI:** 10.3389/fphar.2021.805269

**Published:** 2022-01-13

**Authors:** Fangling Zhang, Xiaolin Xiao, Yong Li, Hefei Wu, Xinyu Deng, Yinxiao Jiang, Wenwen Zhang, Jian Wang, Xiao Ma, Yanling Zhao

**Affiliations:** ^1^ State Key Laboratory of Southwestern Chinese Medicine Resources, School of Pharmacy, Chengdu University of Traditional Chinese Medicine, Chengdu, China; ^2^ Hospital of Chengdu University of Traditional Chinese Medicine, School of Clinical Medicine, Chengdu University of Traditional Chinese Medicine, Chengdu, China; ^3^ Department of Pharmacy, The Fifth Medical Center of PLA General Hospital, Beijing, China

**Keywords:** GPBAR1, bile acids, cholestasis, liver disease, inflammation, GPBAR1 agonists

## Abstract

GPBAR1, a transmembrane G protein-coupled receptor for bile acids, is widely expressed in multiple tissues in humans and rodents. In recent years, GPBAR1 has been thought to play an important role in bile homeostasis, metabolism and inflammation. This review specifically focuses on the function of GPBAR1 in cholestatic liver disease and summarizes the various pathways through which GPBAR1 acts in cholestatic models. GPBAR1 mainly regulates cholestasis in a holistic system of liver-gallbladder-gut formation. In the state of cholestasis, the activation of GPBAR1 could regulate liver inflammation, induce cholangiocyte regeneration to maintain the integrity of the biliary tree, control the hydrophobicity of the bile acid pool and promote the secretion of bile HCO_3_^−^. All these functions of GPBAR1 might be clear ways to protect against cholestatic diseases and liver injury. However, the characteristic of GPBAR1-mediated proliferation increases the risk of proliferation of cholangiocarcinoma in malignant transformed cholangiocytes. This dichotomous function of GPBAR1 limits its use in cholestasis. During disease treatment, simultaneous activation of GPBAR1 and FXR receptors often results in improved outcomes, and this strategy may become a crucial direction in the development of bile acid-activated receptors in the future.

**GRAPHICAL ABSTRACT:**
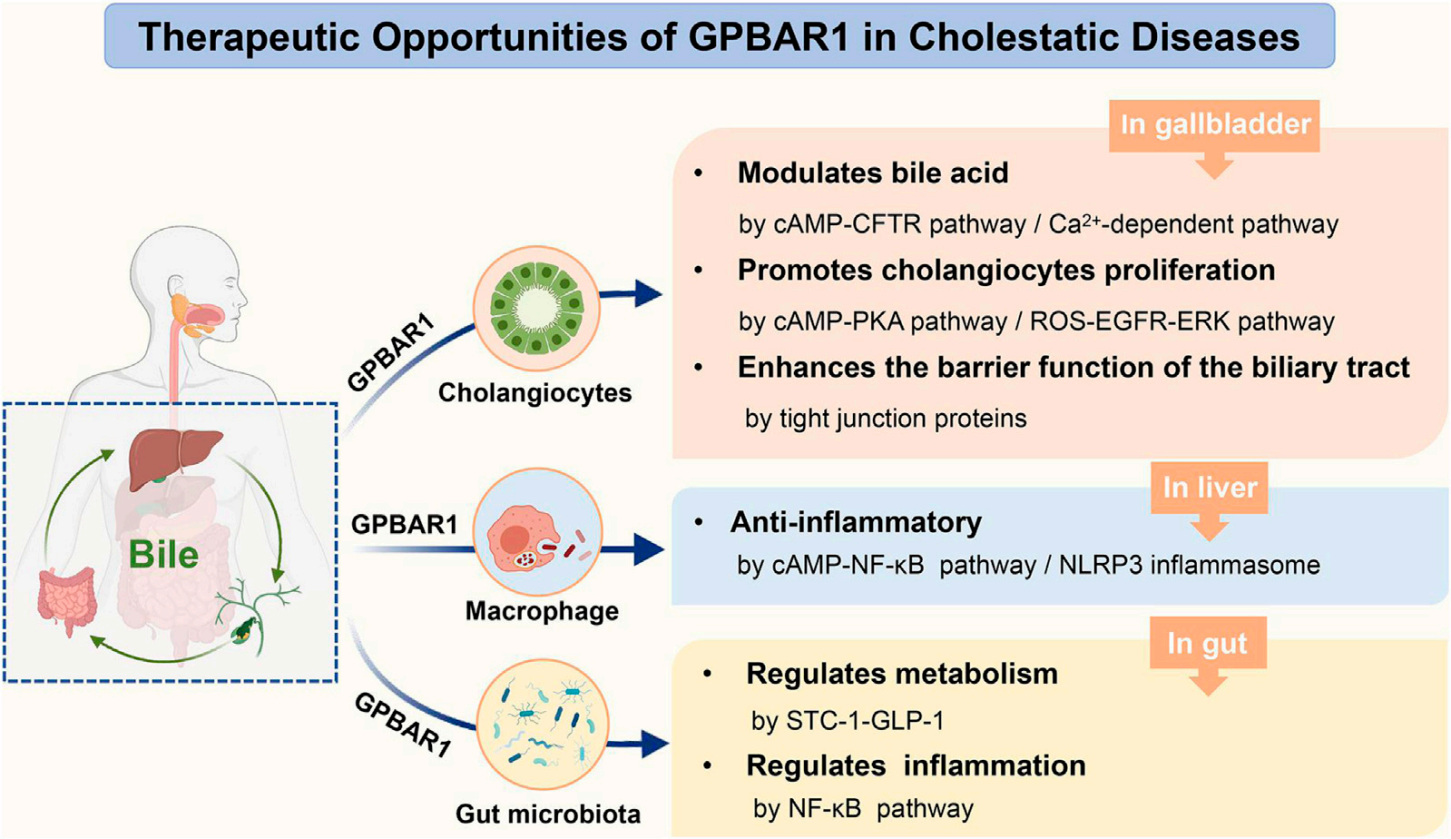
The role of GPBAR1 in cholestatic diseases.

## Introduction

Cholestasis is defined as the abnormal formation and excretion of bile caused by various internal and external factors in the liver, followed by modification of the bile composition, which may be intrahepatic cholestasis or extrahepatic cholestasis. Viruses, alcohol, stones, drugs, autoimmune and genetic metabolic diseases are common causes of cholestasis. Early clinical manifestations of cholestasis are increases in alkaline phosphatase (ALP) and gamma-glutamyl transferase (GGT) levels; then, as the disease develops, symptoms such as jaundice and itching can occur. Severe cholestatic liver disease can lead to hyperbilirubinemia, liver failure, and even death (Chen et al., 2018). The incidence and prevalence of cholestasis have increased globally over the past decades. Cholestasis remains an important public health problem that needs to be effectively addressed. Currently, the most common medical treatment for patients with cholestasis is ursodeoxycholic acid (UDCA), which slows the progression of primary biliary cirrhosis (PBC). However, there are patients who do not respond well to UDCA, and UDCA treatment does not improve survival in primary sclerosing cholangitis (PSC) patients (Ghonem et al., 2015). Due to such limited treatments, alternative therapies are needed. With the development of studies on the aetiology and mechanism of cholestatic liver disease, novel therapies using bile acid (BA)-activated receptors to treat cholestasis have attracted increasing attention.

As the largest superfamily of receptors, G protein-coupled receptors (GPCRs) are involved in almost all aspects of human physiology and myriad disease signalling processes and play essential roles in different cell signalling pathways. Since GPCRs account for approximately 30% of current drug therapeutic targets, these receptors have become one of the most important therapeutic targets for many diseases (Komatsu, 2015). GPBAR1 (also called TGR5, BG37, M-BAR, hGPCR19, and AXOR 109) is a GPCR that was initially discovered in 2002 (Maruyama et al., 2002; Zhong, 2010) GPBAR1 is a transmembrane GPCR that responds to BAs and is widely expressed in various cell types, including cholangiocytes, gallbladder smooth muscle cells and intestinal cells, nerve cells and brown adipose cells (Malhi and Camilleri, 2017; van Nierop et al., 2017). The role of GPBAR1 in metabolic diseases has always been a hot topic of research. As a metabolic regulator, GPBAR1 is involved in the regulation of energy homeostasis and glucose metabolism and has been indicated to have great potential value in the treatment of obesity and type 2 diabetes (Guo et al., 2016a). Furthermore, deoxycholic acid (DCA) and lithocholic acid (LCA), which are natural ligands of GPBAR1, have been shown to have anti-inflammatory effects (Yoneno et al., 2013). Activating GPBAR1 signalling can inhibit the production of proinflammatory cytokines in gastroenteropathy and reduce inflammation in both monocytes and macrophages (Perino and Schoonjans, 2015). To date, the role of GPBAR1 in various physiological and pathological processes has been verified. As various related studies have increased, the role of GPBAR1 in liver disease has also attracted specific attention.

In the liver, GPBAR1 is highly expressed in different nonparenchymal cells, including sinusoidal endothelial cells, Kupffer cells, activated hepatic stellate cells and cholangiocytes, and mediates liver microcirculation, the hepatic inflammatory response and the regulation of biliary function (Keitel and Häussinger, 2018). Cholangiocytes are the main cells that express GPBAR1, which has established the important role of GPBAR1 in biliary secretion, bile duct proliferation and apoptosis as well as other kinds of biliary diseases. In a mouse model of common bile duct ligation (BDL) and BA nourishment, GPBAR1-knockout (KO) mice exhibited more serious liver damage than wild-type (WT) mice, and prolonged cholestasis and an exacerbated inflammatory response were also observed (Péan et al., 2013). This finding provides direct evidence of the potential role of GPBAR1 in the process of cholestatic liver disease, suggesting that GPBAR1 could be an effective target in the treatment of cholestasis.

There is no doubt that the therapeutic efficacy of targeting GPBAR1 in cholestasis has great potential and should be explored. GPBAR1 could protect the liver from BA-induced apoptosis and protect cholangiocytes from BA-induced toxicity. Furthermore, in the pathological state of cholestasis, BA-activated GPBAR1 may mainly affect enterohepatic circulation organs, including the liver, gallbladder and intestine. Since GPBAR1 is widely expressed in these enterohepatic circulation tissues, it tends to regulate cholestasis through a variety of different pathways. In this review, we summarize our understanding of the role of GPBAR1 in the pathophysiology of cholestatic diseases and expound on the different pathways that mediate the effects of GPBAR1 on cholestatic liver disease.

## GPBAR1 Modulates Bile Acid Homeostasis

As the main component of bile, BAs are synthesized in the liver and return to the liver *via* the enterohepatic cycle. During ileal reabsorption, some of the BAs escape into the colon, where they are converted by the intestinal microbiota into secondary BAs. The secondary BA is more hydrophobic and passively interacts with the colon epithelium to form the so-called BA pool with other BAs (Merlen et al., 2020a). Normally, BAs are almost completely confined to enterohepatic circulation, with only trace escapes in the cycle general. The amphiphilic structure of the BA molecule determines whether it is protective or toxic (Portincasa et al., 2020). Hydrophobic BA tends to cause toxic effects, and the stronger the hydrophobicity of the BA pool, the more harmful the impact on liver tissue (Merlen et al., 2020a). Thus, under the pathological condition of cholestasis, regulation is important to prevent excessive hydrophobic BAs from affecting liver repair.

Maruyama et al*.* showed that compared with that of WT mice, the size of the total BA pool in GPBAR1-deficient mice was significantly decreased by 21–25%, suggesting that GPBAR1 contributes to BA homeostasis (Maruyama et al., 2006). GPBAR1 is hardly expressed in hepatocytes, so GPBAR1 may not be directly associated with BA synthesis and tubule bile secretion. Consistent with this finding, there was no significant difference in the mRNA expression of a key enzyme in the conversion of cholesterol to BA (CYP7a1, CYP8b1, CYP27a1) and BA transporters between GPBAR1-KO mice and WT mice before and after partial hepatectomy (PH) (Péan et al., 2013). However, it is expected that high GPBAR1 expression in cholangiocytes may affect the ductal components of bile secretion (Merlen et al., 2020a). Accordingly, Keitel et al. demonstrated that GPBAR1 mediates chloride secretion in biliary epithelial cells by activating cystic fibrosis transmembrane conductance regulator (CFTR) (Keitel et al., 2009). Péan et al. observed the effect of GPBAR1 on protecting hepatocytes and maintaining remnant liver function after PH. The livers of GPBAR1-KO mice accumulated excessive hydrophobic BA pools and exhibited excessive liver inflammation, suggesting that GPBAR1 could control bile hydrophobicity, as well as cytokine secretion under BA overload after PH. Furthermore, Péan et al. also suggested that GPBAR1 regulates ion exchange to provide further protection against BA overload (Péan et al., 2013). GPBAR1 may regulate ion exchange in bile through cAMP-mediated mechanisms at posttranslational steps. This ion exchange process involves the concept of a HCO_3_^−^ umbrella. When cholangiocytes are exposed to high concentrations of hydrophobic BAs, the self-protection mechanism of the cell not only forms micelles but also regulates the pH of the apical membrane by secreting HCO_3_^−^ from the bile to form a HCO_3_^−^ umbrella (Beuers et al., 2010). This alkaline environment prevents the protonation of glycine-conjugated BAs and their uncontrolled penetration into the apical membrane, which can cause liver damage.

**FIGURE 1:**
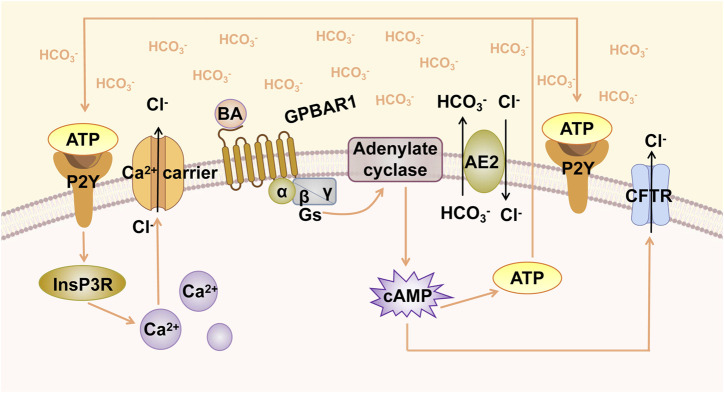
GPBAR1-induced biliary HCO_3_^−^ umbrella. GPBAR1 is activated by BA, leading to the activation of stimulatory G-protein (Gs) and adenylate cyclase, resulting in increased intracellular cAMP levels. cAMP activates CFTR, triggering the secretion of Cl^−^. AE2 mediates the exchange of Cl^−^/HCO_3_^−^ on the apical membrane and promotes the formation of HCO_3_^−^ umbrella. Increased cAMP levels induce the release of ATP, and ATP binds to the P2Y receptor and activates Ca^2+^-dependent chloride channels.

The stability of the HCO_3_^−^ umbrella is associated with GPBAR1-mediated Cl^−^/HCO_3_^−^ exchange and HCO_3_^−^ secretion (Beuers et al., 2010). Hohenester et al. explored the factors that maintain the integrity of human cholangiocytes in millimolar bile salt monomers *in vitro*. The results indicated that bile maintained an alkaline pH in the apical membrane of cholangiocytes through the secretion of HCO_3_^−^. Anion exchanger 2 (AE2) is a Cl^−^/HCO_3_^−^ exchanger that plays a key role in human biliary HCO_3_^−^ secretion (Hohenester et al., 2012). Thus, GPBAR1 participates in the secretion of HCO_3_^−^ through CFTR and AE2 in a cAMP-dependent manner (Keitel et al., 2009; Hohenester et al., 2012). Moreover, a biliary glycocalyx may function to protect bile duct cells from bile salt toxicity; this glycocalyx is present in the outer lobes of the apical membrane of cholangiocytes, which enhances the ability of the bile HCO_3_^−^ umbrella to inhibit the entry of hydrophobic BAs into the cells (Hohenester et al., 2012). The effect of the glycocalyx is similar to that of AE2 and appears crucial for the stability of the GPBAR1-mediated HCO_3_^−^ umbrella. The concept of the biliary HCO_3_^−^ umbrella is also applicable to the pathogenesis of various human fibrotic cholangiopathies. In humans, GPBAR1 has been confirmed to be a susceptibility gene in patients with PSC (Karlsen et al., 2010). When GPBAR1 gene is defective, it may affect the formation of the HCO_3_^−^ umbrella and exacerbate liver injury. The CFTR-dependent chlorine transport pathway mentioned previously for Cl^−^/HCO_3_^−^ exchange is considered to be the most typical pathway for HCO_3_^−^ secretion. In addition, there is a Ca^2+^-dependent HCO_3_^−^ secretion pathway (Nathanson et al., 1996). cAMP acts through an autocrine circuit that involves ATP release, P2Y receptor activation, and cytoplasmic Ca^2+^ increase. ATP release into the lumen stimulates P2Y nucleotide receptors in the apical region of cholangiocytes, which in turn causes an increase in Ca^2+^ (Figure 1). This series of processes leads to cAMP-induced HCO_3_^−^ secretion (Minagawa et al., 2007). Furthermore, loss of inositol 1,4,5-trisphosphate receptor (InsP3R) expression can be observed in patients with cholestasis, such as PBC and PSC patients (Shibao et al., 2003). Although it is not clear whether the loss of InsP3R is the result or the cause of cholestasis, studies have shown that secretion mediated by Ca^2+^ signalling depends on the expression of InsP3R, and the loss of InsP3R reduces the secretion of HCO_3_^−^ (Minagawa et al., 2007). Since the specific regulatory role of GPBAR1 in Ca^2+^ signalling is not yet clear, the relationship between GPBAR1 and InsP3R cannot be thoroughly described. If Ca^2+^-dependent HCO_3_^−^ secretion is determined to play a necessary role in cholestasis, then InsP3R deficiency may affect the efficacy of GPBAR1 to some extent.

There have been few studies on the role of GPBAR1 in the kidney. In the context of BA overload, inadequate elimination of BA in urine exacerbates liver injury after BDL. GPBAR1 may at least promote the excretion of BA in urine by controlling the multidrug resistance-associated protein 2 (MRP2) and MRP4 genes (Péan et al., 2013). In another study, researchers demonstrated that GPBAR1 can regulate the gene and protein expression of aquaporin-2 (AQP2) and intracellular transport through the cAMP-protein kinase A (PKA) pathway and affect the transmembrane transport of water in the kidney. Moreover, glycogen synthase kinase 3β (Gsk3β) may be the downstream target of the GPBAR1 signalling pathway (Li S. et al., 2018). GPBAR1 may have a similar water-regulating role in the biliary epithelium. In line with this hypothesis, GPBAR1 can regulate the distribution of BAs through the selective reabsorption of hydrophobic BAs by the biliary epithelium, which is a process known as cholehepatic shunting (Holter et al., 2020). Specifically, GPBAR1 can promote the insertion of apical sodium-dependent BA transporter (ASBT) into the parietal membrane through cAMP and enhance BA uptake by biliary epithelial cells (Keitel et al., 2009). Cholehepatic shunting is thought to limit the hydrophobicity of the BA pool through the reabsorption of secondary BA by biliary epithelial cells.

In addition, early reports also pointed out that BAs can cause the release of nitric oxide (NO) through the GPBAR1 receptor (Merlen et al., 2020a). In sinusoidal vascular endothelial cells exposed to high concentrations of BA, GPBAR1 can activate and regulate the production of NO through cAMP-dependent endothelial nitric oxide synthase (eNOS); this mechanism that can scavenge BA-induced ROS is also of importance in the protection of the liver parenchyma (Thomas et al., 2008).

## The Dichotomous Pro-Proliferative Effect of GPBAR1 in Cholangiocytes

### GPBAR1 Maintains the Integrity of the Biliary Tree

Cholangiocytes are a group of epithelial cells and highly specialized cells which line the biliary epithelium; these cells were once considered to be a type of dormant cell but are now considered to be active and hormone-responsive cells (Jones et al., 2015; Banales et al., 2019). During the process of bile duct disease, cholangiocytes respond to exogenous and endogenous damage and directly participate in the progression of bile duct disease; thus, cholangiocytes are considered to be the targets of chronic liver disease in the context of bile duct disease (Guicciardi et al., 2020). Proliferation is a cholangiocyte response that maintains the integrity of the biliary tree during liver injury. Cholangiocyte proliferation could be observed in acute obstructive cholestasis and the early stages of chronic cholestatic liver diseases in humans (Alvaro et al., 2000; Alpini et al., 2002; Lazaridis et al., 2004). Promoting the proliferation of cholangiocytes can benefit the treatment of cholangiopathies, especially PSC and PBC.

**FIGURE 2:**
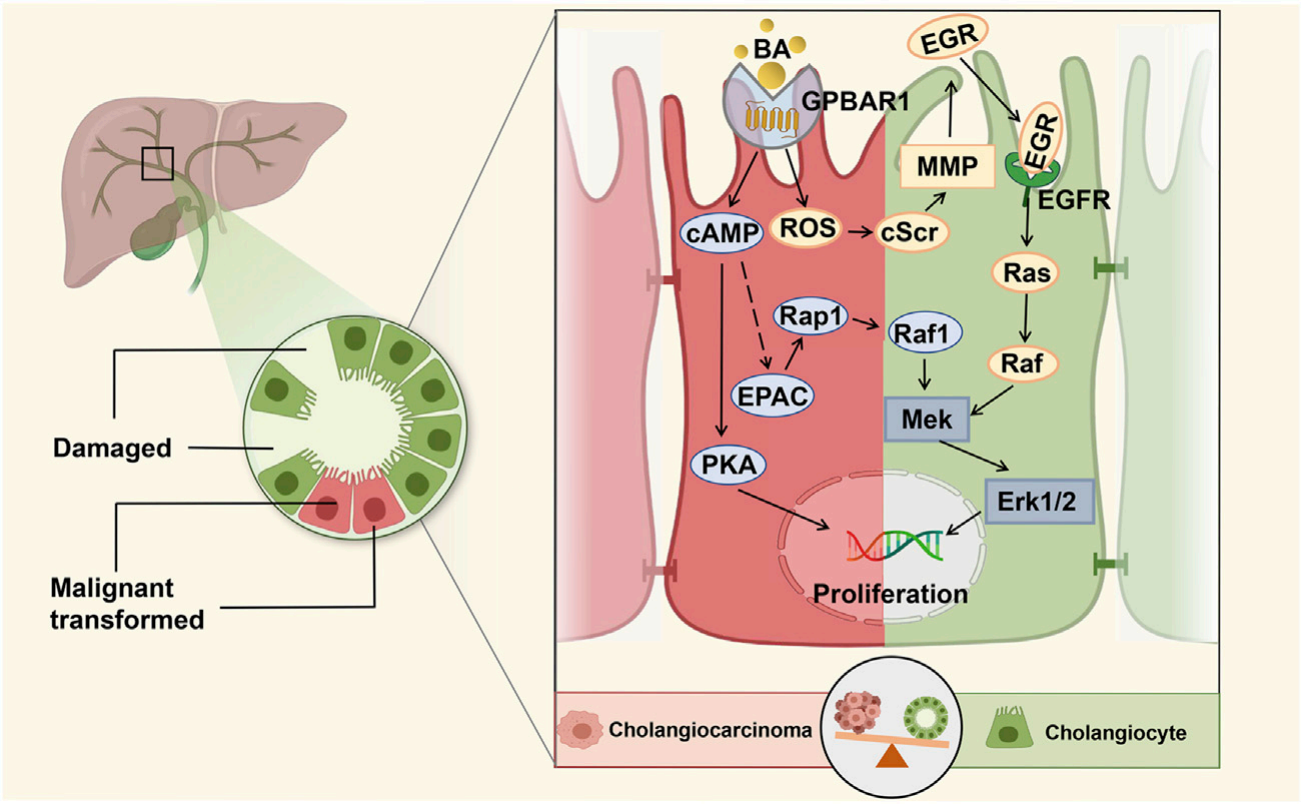
GPBAR1 promotes the proliferation of cholangiocyte. GPBAR1 promotes the proliferation of normal cholangiocytes and cholangiocarcinoma cells through a similar mechanism. GPBAR1 mainly depends on the cAMP pathway to play a proliferative role, and PKA and EPAC are two important downstream effectors of cAMP-induced proliferation. In addition, GPBAR1 can also induce proliferation through an increase in ROS and the activation of EGFR.

GPBAR1 is mainly localized in the apical membrane of gallbladder epithelial cells and primary cilia (Keitel et al., 2009; Keitel and Häussinger, 2012). Several studies have shown that GPBAR1 can promote the proliferation of cholangiocytes, indicating that GPBAR1 can help reduce liver damage under cholestatic conditions in certain circumstances. The intracellular cAMP signalling pathway is responsible for the transmission of BA signals to mediate the cellular functional response. When GPBAR1 is activated, it leads to an increase in the level of intracellular cAMP, followed by an associated cell-specific response (Kawamata et al., 2003; Keitel and Häussinger, 2011). GPBAR1 is linked to cAMP and expressed in cholangiocytes, and cholangiocyte proliferation is closely associated with intracellular cAMP levels (Sand et al., 1992; Lesage et al., 1996; Alpini et al., 1997b). Therefore, GPBAR1 seems to be able to induce cell proliferation through the cAMP pathway (Figure 2). In early experiments, Alpini et al. showed that the effects of both up and downregulation of BAs on cholangiocyte proliferation and secretion are associated with the synthesis of cAMP in cholangiocytes (Alpini et al., 1999). In another study, the researchers mentioned that micromolar concentrations of BA could be used as signals for secretory and proliferative events in large but not small cholangiocytes, while small cholangiocytes may exhibit the phenotype of large cholangiocytes when they proliferate (Alpini et al., 1997a). The responses of large and small cholangiocytes are different in all kinds of bile duct injury experiments, but their responses to BAs are similar (Sato et al., 2018). These studies showed that cAMP is a key messenger for cholangiocyte proliferation. PKA and exchange proteins activated by cAMP (EPACs) may be two important downstream effectors of cAMP-induced cholangiocyte proliferation (Banales et al., 2009). PKA is one of the most well-studied serine/threonine protein kinases and has been reported to be involved in cAMP-mediated proliferation in multiple cell types (Hogarth et al., 2004; Masyuk et al., 2017; Meroni et al., 2019; Smith et al., 2019). The increase in cAMP may activate PKA expression, which in turn promotes cell proliferation. Additionally, two new PKA-independent cAMP effectors, EPAC1 and EPAC2, have also been described to have a variety of cAMP-mediated biological effects including cell proliferation. It should be noted that the activation of EPAC has a cell type-specific effect on proliferation, inducing growth inhibition in vascular smooth muscle cells and growth promotion in other cell types, including endothelial cells (Smith et al., 2019). Therefore, GPBAR1 may induce cholangiocyte proliferation through the cAMP/PKA pathway, while EPAC may participate in regulating proliferation through a signalling pathway parallel to or independent of PKA. Regarding the conditions for cell proliferation, early studies also emphasized the effect of apical BA transporter (ABAT) on the effects of BAs on cholangiocytes. Researchers suggested that BAs can change the function of cholangiocytes only in the presence of sodium *in vitro* (Alpini et al., 1999). When this sodium-dependent transporter takes up BAs from bile, it may initiate signals to modulate cholangiocyte proliferation and secretion. Recent research on sodium-dependent transporters in cholestasis models has focused on ASBT, and pharmacological ASBT inhibition could attenuate cholestatic liver and bile duct injury by reducing biliary BA concentrations in mice (Baghdasaryan et al., 2016). Whether the existence of such sodium-dependent transporters is conducive to the proliferation of cholangiocytes thus contributes to the treatment of cholestasis seems to be no longer the focus of modern research.

In a previous study, researchers found that ERK activation in mouse cholangiocytes was a downstream signal of BA-induced cholangiocyte proliferation (Francis et al., 2004). Similarly, in a high-quality study, Reich et al. demonstrated that cholangiocyte proliferation was significantly decreased in GPBAR1-KO mice compared with WT mice under the pathological condition of cholestasis. GPBAR1 induced cholangiocyte proliferation *via* increased reactive oxygen species (ROS) and the activation of epidermal growth factor receptor (EGFR) and ERK1/2. The specific proliferation pathway can be summarized as the GPBAR1-ROS-Src-MMP-EGFR-ERK-dependent signalling pathway, which indicates that GPBAR1 can trigger cell proliferation independent of adenylate cyclase activation (Reich et al., 2016). Enhancing the barrier function of the biliary tract is an important regulatory mechanism to protect liver cells from the toxic damage induced by BA in cholestatic liver disease. Merlen et al. focused on the relationship between GPBAR1 and tight junction proteins (TJPs), and the experimental results showed that liver injury caused by cholestasis in GPBAR1-KO mice was more serious than that in WT mice. The protective effect of the GPBAR1 agonist on cholestasis-induced liver injury was significantly weakened in JAM-A-KO mice, and GPBAR1 affected the expression and phosphorylation of the main TJP JAM-A to regulate biliary epithelial barrier function. PKCζ is a major kinase involved in JAM-A phosphorylation, which is at least partially activated in the course of JAM-A phosphorylation (Merlen et al., 2020b).

It is worth noting that different localizations of GPBAR1 in cholangiocytes may produce different biological effects. Masyuk et al. showed that GPBAR1 agonists caused opposing changes in cAMP and ERK levels in ciliated and nonciliated H69 cells. GPBAR1 agonists activated the proliferation of nonciliated cells but not ciliated cholangiocytes. This difference may be associated with the coupling of GPBAR1 with the Gαs protein in nonciliated cells and the Gαi protein in ciliated cells (Masyuk et al., 2013). In general, the subcellular localization of GPBAR1 directly affects the functional response of cholangiocytes to BA signals.

### GPBAR1 Promotes the Proliferation of Cholangiocarcinoma Cells

Owing to the nearly ubiquitous expression of GPBAR1 in human cells, GPBAR1 shows dichotomous functions in different diseases. As previously mentioned, GPBAR1 activation promotes cholangiocyte proliferation and effectively protects cholangiocytes from BA-induced toxicity in the context of cholestasis. However, GPBAR1 activation can change from beneficial to detrimental in the case of malignant transformed cholangiocytes and promote the risk of cholangiocarcinoma (CCA) proliferation. CCA is a malignant epithelial cell tumour originating from varying locations within the biliary tree and is characterized by the differentiation of cholangiocytes (Razumilava and Gores, 2014). The aetiology of CCA is diverse; here, we focus on the association between cholestasis and CCA. It was reported that CCA may be the consequence of long-term cholestasis and inflammation in the liver (Erice et al., 2018). Most risk factors for CCA can cause cholestasis or chronic inflammation (Labib et al., 2019). Once patients with cholestasis develop CCA at a later stage, the activation of the BA receptor GPBAR1 may exacerbate the situation.

Studies have shown that bile salts facilitate the development of CCA by inducing biliary proliferation, promoting liver inflammation, downregulating FXR and upregulating GPBAR1 (Deutschmann et al., 2018). The expression of GPBAR1 in CCA has been indicated to be increased, and GPBAR1 activation in human CCA cells promotes cell proliferation *via* a mechanism similar to that in mouse cholangiocytes. GPBAR1 was activated in a CCA cell line, induced the phosphorylation of EGFR and ERK1/2, and further promoted cell proliferation (Reich et al., 2016). At present, there are no clinical data showing that the expression of GPBAR1 is associated with the pathology of CCA patients. In view of this, Li et al. measured the expression of GPBAR1 in 20 pairs of extrahepatic cholangiocarcinoma (ECC) specimens and paratumoural tissues and demonstrated that GPBAR1 was highly expressed in CCA tissues. In addition, GPBAR1 promoted the proliferation, migration and apoptosis resistance of CCA cells. Furthermore, it was observed that GPBAR1 could bind to mortalin and regulate its expression in the CCA cell line; mortalin may be a downstream component of GPBAR1 that promotes CCA cell proliferation, and the interaction between GPBAR1 and mortalin may at least partially promote the occurrence of CCA (Li et al., 2020). Therefore, in the context of CCA, inhibiting GPBAR1 expression at least partially inhibits the proliferation and migration of cancer cells, indicating that GPBAR1 may be a potential therapeutic target for CCA treatment. It is important to note that this effect is the exact opposite of what GPBAR1 needs to do in cholestasis, and the late stage of cholestasis may be the key point for the functional transformation of GPBAR1.

## GPBAR1 Mediates the Inhibition of Liver Inflammation

The cause of cholestasis has been widely studied, but the mechanism of liver injury caused by BA is still unclear. BA at the submillimolar level is highly toxic and can directly damage hepatocytes, but even under pathological conditions, toxic BA rarely reaches this submillimolar level; a certain concentration of glycyldeoxycholic acid (GCDCA) can cause apoptosis in rat hepatocytes, but serum GCDCA cannot reach this concentration even in the case of complete biliary obstruction (Li M. et al., 2017). These results suggest that BA-induced liver injury may occur through alternative mechanisms. Recent studies have shown that excess BA is not directly cytotoxic to hepatocytes. During cholestasis, BA, as an inflammatory factor, activates the signalling pathway in hepatocytes and increases the expression of proinflammatory mediators, indicating that BA-mediated proinflammatory effects are the key to causing cholestatic liver injury (Allen et al., 2011; Cai et al., 2017).

The expression of chemokines by hepatocytes is the early initiation event of inflammatory pathogenesis during cholestasis. These specific cytokines can significantly enhance neutrophil chemotaxis and play a crucial role in triggering inflammation (Li M. et al., 2017). In early studies, hydrophilic BAs could not activate GPBAR1, nor could they regulate macrophage activity. Only hydrophobic BAs such as TLC, TC, GCDC, and TCDC have effects on macrophage activation. These BAs are known ligands of GPBAR1, suggesting that BAs may mediate lipopolysaccharide (LPS)-induced inflammation by activating GPBAR1 (Kawamata et al., 2003). In the GPBAR1-KO mouse model, additional direct evidence could be obtained regarding whether GPBAR1 mediates liver inflammation. In the LPS-induced inflammation model, GPBAR1-KO mice showed more severe liver injury and inflammation than WT mice (Wang et al., 2011). Activation of GPBAR1 inhibited the expression of cytokines in mouse macrophages and rat Kupffer cells induced by LPS (Keitel et al., 2008; Wang et al., 2011). GPBAR1 plays the same role in human macrophages (Haselow et al., 2013). GPBAR1 gene KO increased proinflammatory mediators and neutrophil transport in BDL-induced cholestatic mice (Rao et al., 2020). These related studies have shown the anti-inflammatory and hepatoprotective effects mediated by GPBAR1. Considering that GPBAR1 is hardly expressed in hepatocytes but is enriched in macrophages and Kupffer cells, GPBAR1-dependent anti-inflammatory effects during cholestasis may occur downstream of BA-mediated hepatocyte inflammation (Merlen et al., 2020a).

**FIGURE 3:**
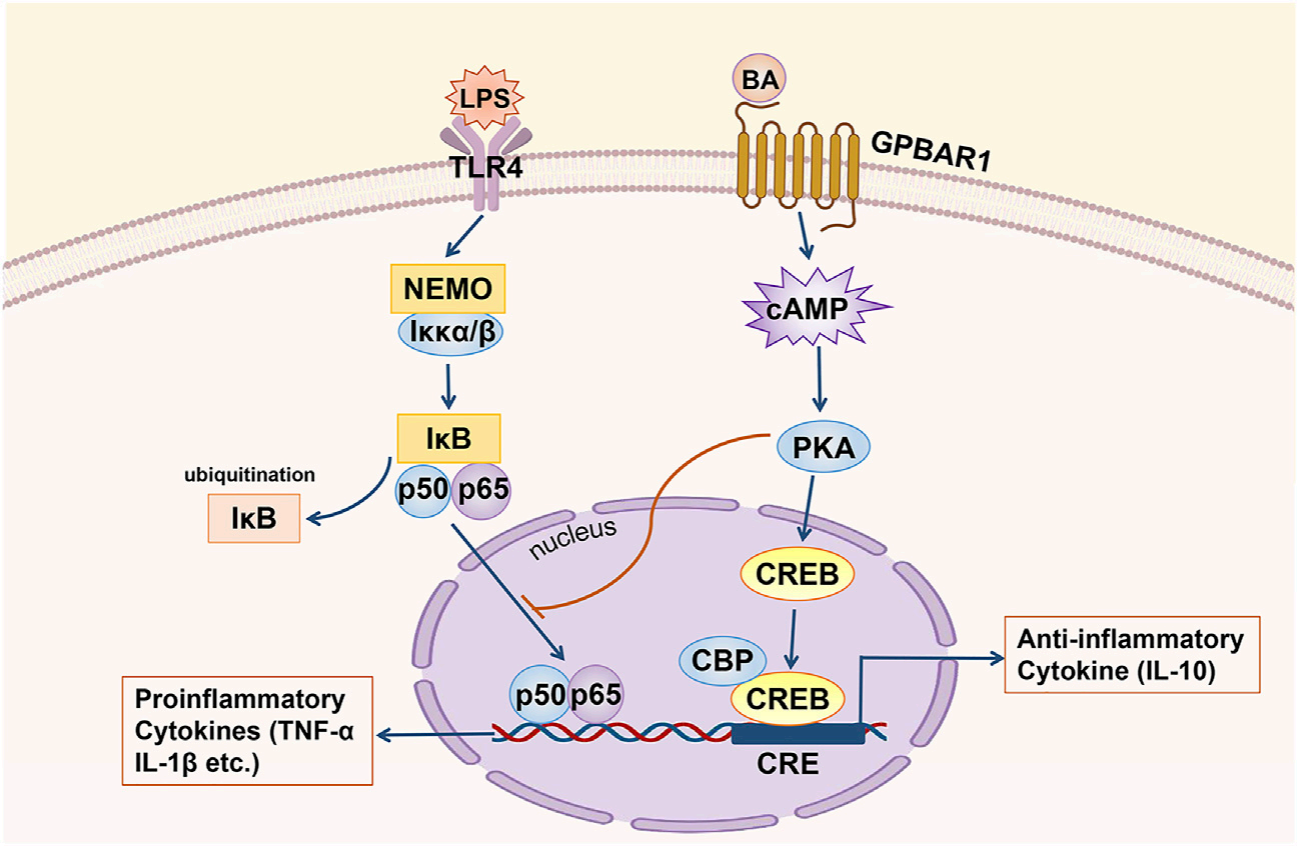
GPBAR1 relieves hepatic inflammation. GPBAR1 through the cAMP pathway inhibits the transcription of proinflammatory cytokines mediated by NF-κB (IL-1α, IL-1β and TNF-α, etc.) and maintains the expression of an anti-inflammatory cytokine (IL-10).

Regarding the relevant mechanism by which GPBAR1 mediates anti-inflammatory in cholestasis, in the macrophages of GPBAR1-KO mice, the RNA levels of various proinflammatory genes, such as inducible nitric oxide synthase (iNOS), interferon-inducible protein and IL-1, which are targeted by NF-κB, were higher than those of WT mouse macrophages, suggesting that the anti-inflammatory effect of GPBAR1 is mediated by inhibiting NF-κB (Calmus and Poupon, 2014) (Figure 3). Wang et al. indicated that GPBAR1 inhibited the NF-κB pathway by mediating the interaction between Iκbα and β-arrestin2 and that GPBAR1 was a negative regulator of NF-κB-mediated liver inflammation (Wang et al., 2011). However, in another study, the role of β-arrestin2 was not confirmed. Pols et al. used β-arrestin2-KO cells to show that the β-arrestin2 molecule cannot be coupled with the anti-inflammatory effect induced by GPBAR1. In macrophages treated with GPBAR1 agonists, the inhibition of cytokine production depends on activation of the cAMP-NF-κB signalling pathway (Pols et al., 2011). cAMP inhibits NF-κB activity, thus effectively inhibiting liver inflammation. Keitel et al*.* demonstrated that GPBAR1 was located in the plasma membrane of Kupffer cells. BA-induced activation increases the production of cAMP and inhibits the expression of proinflammatory cytokines including IL-1α, IL-1β, IL-6 and TNF-α, induced by LPS (Keitel et al., 2008). The activation of GPBAR1 in macrophages reduces the production of proinflammatory cytokines and maintains the expression of anti-inflammatory cytokines, thereby promoting the development of an anti-inflammatory macrophage phenotype (Li M. et al., 2017). The key anti-inflammatory effects of cAMP are specifically mediated by cAMP-dependent PKA (Wall et al., 2009). In primary human macrophages, inhibition of PKA with a specific inhibitor resulted in an almost complete reduction in TLC-mediated inhibition of LPS-induced cytokine expression. GPBAR1 is at least partially involved in regulating the BA-mediated response to LPS-induced inflammation. This series of processes involves BAs activating GPBAR1, increasing the production of cAMP and the subsequent activation of PKA (Haselow et al., 2013). On the other hand, the PKA-dependent enhancement of LPS-induced anti-inflammatory cytokine (IL-10) expression depends on the presence of CREB (Wall et al., 2009). In LPS-induced inflammation, rescuing inflammatory cytokine expression through the inhibition of PKA is associated with the suppression of BA-induced CREB activation. CREB activation has been shown to inhibit the transcriptional activity of NF-κB by competing with NF-κB for the transcriptional coactivator CBP (Haselow et al., 2013). The upregulation of IL-10 by cAMP may be an important mechanism by which cAMP inhibits the activation of phagocytes. However, cAMP can also inhibit the production of TNF-α and IL-12 by IL-10-deficient dendritic cells (DCs), so there may be another important cAMP-mediated mechanism that inhibits the expression of LPS-induced inflammatory factors (Koga et al., 2009). The transcription factor c-Fos accumulates massively under the stimulation of cAMP and LPS; when the expression of c-Fos is enhanced, c-Fos physically interacts with the p65 protein and inhibits NF-κB-mediated gene expression (Koga et al., 2009; Haselow et al., 2013). Hence, BAs can inhibit the expression of LPS-induced proinflammatory factors through two different cAMP-driven mechanisms. Furthermore, after treatment with BA, the LPS-induced nuclear translocation of NF-κB was delayed. The inhibitory effect of this nuclear translocation is also PKA-dependent, showing a mutual connection with the first two mechanisms (Haselow et al., 2013). In addition, Ichikawa et al. indicated that specific GPBAR1 agonists can induce DCs to exhibit an IL-12 hypoproducing phenotype, resulting in DC differentiation into cells with an anti-inflammatory phenotype. GPBAR1 is downregulated rapidly during the differentiation of monocytes into DCs, and 8-Br-cAMP, which is downstream of the signal, responds to this downregulation, which stimulates and promotes the differentiation of DCs into the IL-12 hypoproducing phenotype (Ichikawa et al., 2012). This process is cAMP-dependent.

Inflammasomes assist host defence against pathogens and play key roles in inflammation (Davis et al., 2011). Previous studies have extensively investigated the NLRP3 inflammasome. The association between GPBAR1 and NLRP3 has also been demonstrated, but most studies have mainly confirmed the effect of GPBAR1 on the NLRP3 inflammasome in the context of neuroinflammation, colitis, pancreatitis and nonalcoholic steatohepatitis (NASH) (Li B. et al., 2018; Chen et al., 2019; Shi et al., 2020; Liang et al., 2021). Few studies have explored the combined effects of GPBAR1 and inflammasomes in cholestasis. Researchers have shown that BA can inhibit the activation of NLRP3 through the GPBAR1-cAMP-PKA axis. GPBAR1-induced PKA activation causes NLRP3 ubiquitination and phosphorylation, while NLRP3 phosphorylation plays a crucial role in the inhibition of NLRP3 inflammasome activation (Guo et al., 2016b). However, scholars have shown that the activation of GPBAR1 activates the NLRP3 inflammasome, causing inflammation in the liver during cholestasis. This effect is contrary to the results of previously mentioned studies, reflecting the controversial role of GPBAR1 activation in inflammation (Gong et al., 2016).

β-catenin signalling has been shown to be necessary to control innate and adaptive immunity during inflammation (Ke et al., 2013). Rao et al. investigated the relationship between GPBAR1 and β-catenin in cholestatic liver injury. The results showed that by interacting with Gsk3β, GPBAR1 activated the β-catenin signalling pathway to control local liver inflammation in immune-mediated cholestatic liver injury. GPBAR1-mediated β-catenin signalling also activated PI3K/AKT and inhibited the TLR4/NF-κB pathway, thus attenuating the inflammatory response, which suggests a new mechanism by which GPBAR1 regulates cholestatic liver disease in a model of cholestatic liver injury induced by BDL (Rao et al., 2020).

## GPBAR1 Regulates Intestinal Metabolism and Inflammation

After the body ingests food, microbiota converts polysaccharides to short-chain fatty acids and generates amino acids and metabolites, which can be used by the host to harvest energy. Metabolites send signals through their cognate receptors to regulate the metabolism of the host; thus, the gut microbiota is considered a metabolic organ (Wahlström et al., 2016). There is a close relationship between the liver and the gut, which is known as the gut-liver axis. Seventy percent of the blood supply of the liver flows directly from the veins of the intestine (Li Y. et al., 2017). BAs, which are synthesized from cholesterol in the liver and further metabolized by the gut microbiota, are a class of important metabolites that are produced microbially and some of the most abundant metabolites in the intestine (Sorrentino et al., 2020). Evidence has shown that there is an active interaction between the gut microbiota and BAs that plays an important role in the pathogenesis of cholestatic liver diseases such as PBC and PSC (de Aguiar Vallim et al., 2013).

The diversity and richness of the gut microbiota in patients with PSC and PBC were significantly lower than those in healthy individuals (Rossen et al., 2015; Lv et al., 2016). Another study showed that the microbial diversity in PBC patients was significantly decreased, and this effect was reversed after UDCA treatment (Tang et al., 2018). Although the results of current studies on the gut microbiota in PBC and PSC patients are not consistent in terms of changes in species or genera, it is undeniable that the composition of the gut microbiota of patients with cholestasis has changed. On the other hand, studies have shown that the lack of intestinal microbiota can exacerbate hepatobiliary disease in PSC mouse models, indicating the potential protective effect of commensal microbiota against biliary tract injury (Tabibian et al., 2016). Since GPBAR1 is also distributed in the intestine and GPBAR1 regulates cholestasis, is there a close relationship between GPBAR1 and the intestinal microbiota? Does the intestinal microbiota act as another channel through which GPBAR1 regulates cholestasis?

The previous section summarized the role of GPBAR1 in regulating BA homeostasis, cell proliferation and inflammation. In addition, GPBAR1 is also involved in the regulation of metabolism in the body, which seems to connect GPBAR1 and the gut microbiota. Due to the wide diversity of GPCRs and associated ligands, these receptors are almost certainly the basis of the observed ability of the enteroendocrine axis to respond to a wide range of ingested food and luminal components (Gribble and Reimann, 2019). Katsuma et al. showed that BAs promote the secretion of glucagon-like peptide-1 (GLP-1) through GPBAR1 in the enteroendocrine cell line STC-1 (Katsuma et al., 2005). GLP-1 is a pleiotropic hormone with countless metabolic functions (Müller et al., 2019). In terms of intestinal metabolism, studies have shown that diet-induced intestinal microbial dysbiosis in mice is associated with an impaired GLP-1 metabolic response (Grasset et al., 2017). GLP-1 has shown beneficial effects in the treatment of type 2 diabetes and obesity (Gribble and Reimann, 2019). An important study demonstrated that FXR-mediated modification of the intestinal microbiota led to GPBAR1-induced remodelling of the LCA-enriched BA pool and improved glucose tolerance through intestinal GLP-1 secretion. The GPBAR1-GLP-1 axis plays a key role in mediating intestinal BA receptor signal transduction and regulating liver metabolism and homeostasis (Pathak et al., 2018).

Studies have shown that accumulation of BAs in the liver does not contribute to liver damage in the absence of microbiome *in vivo*, suggesting that gut microbiome is critical to the occurrence of cholestasis (Isaacs-Ten et al., 2020). In fact, there is increasing evidence supporting a close relationship between cholestatic liver injury and gut microbiota. However, the role of gut microbiota in the pathogenesis of cholestasis remains unclear. After BDL in mice, the abundance of an anti-inflammatory gut microbiota (*F. prausnitzzi*) was significantly reduced, which may contribute to increased intestinal permeability and loss of intestinal barrier function (Cabrera-Rubio et al., 2019). Isaacs-Ten et al. demonstrated that macrophages contribute to promoting intestinal permeability during cholestasis and change the composition of intestinal microorganisms through activation of inflammasome, aggravating cholestatic liver injury (Isaacs-Ten et al., 2020). And in humans, PSC and inflammatory bowel disease (IBD) co-exist frequently, showing significant correlation. All these suggest that the regulation of intestinal inflammation may be another important way to alleviate cholestatic liver injury.

The anti-inflammatory effect of GPBAR1 mediated in macrophages has been discussed above. Considering the high expression of GPBAR1 in the intestine, GPBAR1 seems to be able to improve cholestasis by regulating intestinal inflammation. In the gastrointestinal tract, the anti-inflammatory effect regulated by macrophages was mainly demonstrated by the activation of GPBAR1, and the activation of GPBAR1 significantly inhibited the activation of NF-κB induced by LPS in WT mice. However, it is still important to note that the role of GPBAR1 in gastrointestinal carcinogenesis has not been determined. There are two types of tumor-associated macrophages: M1 macrophages and M2 macrophages. M1 phenotype produces proinflammatory cytokines (IL-1β, IL-6, IL-12 etc.), while M2 has immunosuppressive effect. Macrophages with mixed phenotype of M1/M2 were induced by GPBAR1 activated by bile acid in macrophages. According to the polarization of macrophages, activation of GPBAR1 in colorectal cancer promotes proinflammatory response in M1 macrophages and anti-inflammatory response in M2 macrophages. The activation of GPBAR1 seems to control the balance of proinflammatory and anti-inflammatory factors in the intestine, and the ratio of IL10:IL12 is an important indicator of intestinal mucosal inflammation (Jia et al., 2018).

## GPBAR1 Agonists

### Natural Bile Acids and Semisynthetic Bile Acid Derivatives

Cholesterol in the liver synthesizes two main primary BAs, cholic acid (CA) and chenodeoxycholic acid (CDCA). And intestinal microbiota converts primary BAs to secondary BAs such as deoxycholic acid (DCA) and lithocholic acid (LCA). Different kinds of BAs have different abilities to activate GPBAR1. These BAs that can activate GPBAR1 may be called the most effective natural agonist of GPBAR1. DCA and LCA are physiological ligands of GPBAR1 (Maruyama et al., 2002). Studies have indicated that the order of activation of GPBAR1 by BA is LCA>DCA>CDCA>CA *in vitro*. In addition, in Chinese hamster ovary cells transfected with human GPBAR1, the order of activation of GPBAR1 by BA is TLCA>LCA>GLCA>TDCA>DCA>GDCA>TCDCA>CDCA>GCDCA>TCA>CA> GCA (Jia et al., 2018). UDCA is a safe and available BA and the mainstream drug for cholestatic liver disease. Studies have shown that UDCA can effectively activate GPBAR1 and play a beneficial role in a NASH mouse model. The EC_50_ of UDCA is 14 μM, which is 4–7 times higher than the TLCA EC_50_ (Carino et al., 2019). TUDCA is a conjugate derivative of UDCA that is commonly used in the treatment of gallstones and chronic cholestatic liver disease (Elia et al., 2016). It has also been shown to activate GPBAR1 and induce an increase in the level of cAMP in microglia, thereby exerting an anti-inflammatory effect (Yanguas-Casás et al., 2017).

**FIGURE 4:**
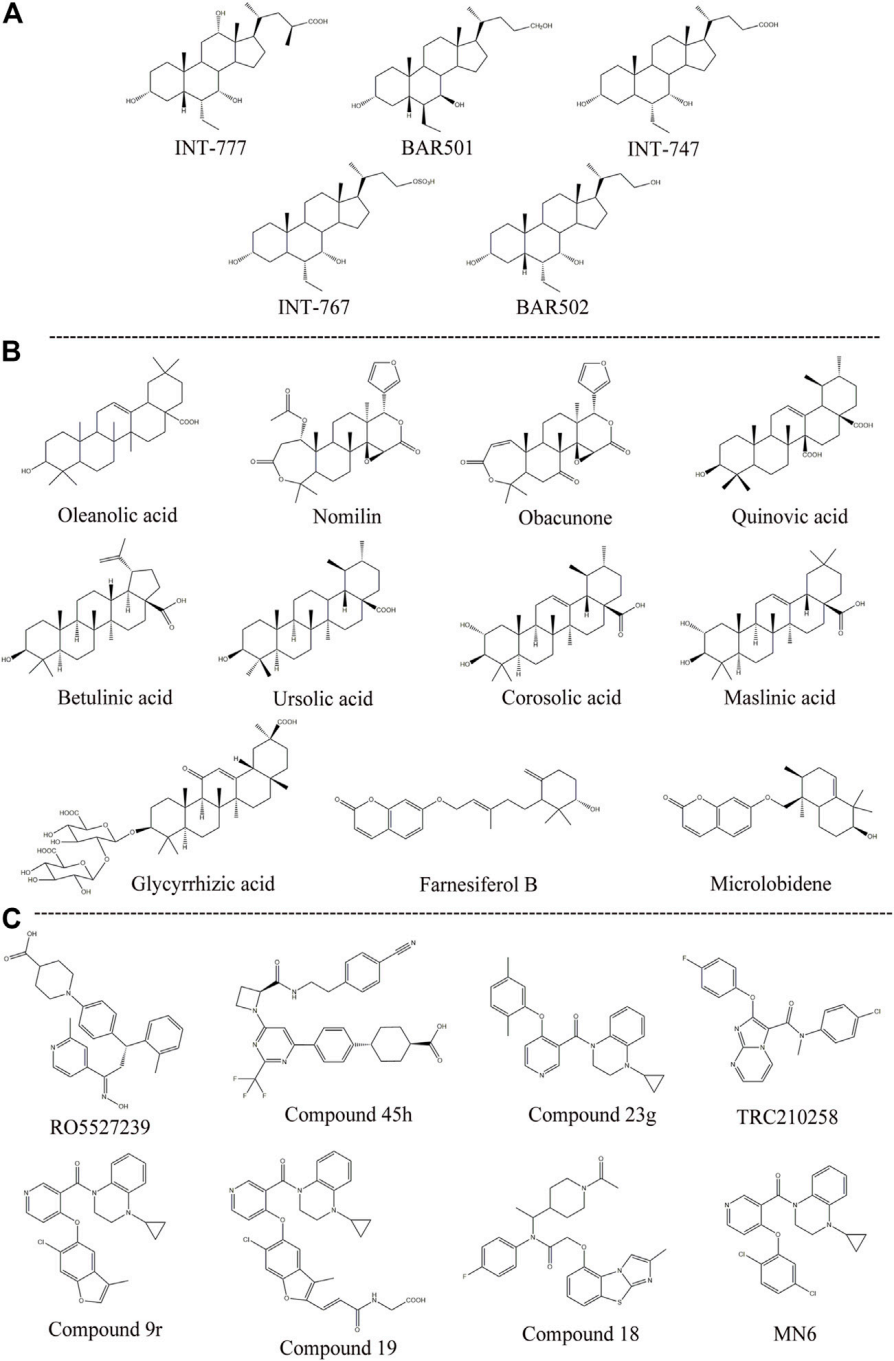
Structures of some typical GPBAR1 agonists. **(A)** Semisynthetic BA derivatives; **(B)** Natural ligand; **(C)** Small molecule.

Semisynthetic BA derivatives can be divided into two kinds of body modifications and side chain modifications (Figure 4; Table 1). INT-777 (6R-ethyl-23(S)-methylcholic acid, S-EMCA, MW 450.65) is a specific GPBAR1 agonist that is widely used in current research and was first described in 2009 (Pellicciari et al., 2009). Studies have shown that GPBAR1 activation by INT-777 can reduce neuroinflammation, improve cognitive impairment, improve glucose tolerance, and increase insulin synthesis (Kumar et al., 2016; Jin et al., 2021). In addition, INT-777 induces the release of GLP-1 from enteroendocrine L-cells, which can improve liver and pancreatic function in mice, stimulate bile flow, and inhibit macrophage inflammation (Thomas et al., 2009; Pols et al., 2011). In addition to INT-777, which is the most commonly used derivative, other semisynthetic derivatives of BA are also being studied. For example, 7ξ-Me-LCA, 7α-F-LCA and CDC-Sul, which were screened from natural BAs, semisynthetic BA derivatives and steroid hormones, are considered to be highly effective and selective GPBAR1 agonists (Sato et al., 2008). BAR501 was the first UDCA derivative with potent and selective GPBAR1 activity (Festa et al., 2014; De Marino et al., 2019). As a small molecular agonist of GPBAR1, BAR501 has been shown to regulate the M1/M2 phenotype of intestinal macrophages and effectively reduce the expression of inflammatory factors (Biagioli et al., 2017).

**TABLE 1:** GPBAR1 agonists and typical GPBAR1/FXR dual agonists.

Compounds	Type	EC50 (μM)	References
INT-777	Semi-BA	0.82	Pellicciari et al. (2009)
7ξ-Me-LCA	Semi-BA	0.076	Sato et al. (2008)
7α-F-LCA	Semi-BA	0.25	Sato et al. (2008)
CDC-Sul	Semi-BA	0.44	Sato et al. (2008)
BAR501	Semi-BA	1.03	Festa et al. (2014)
INT-747	Semi-BA(d)	0.9	De Marino et al. (2019)
INT-767	Semi-BA(d)	0.63	Rizzo et al. (2010)
BAR502	Semi-BA(d)	0.4	De Marino et al. (2019)
Oleanolic acid	Natural ligand	1.42	Sato et al. (2007)
Nomilin	Natural ligand	—	Ono et al. (2011)
Obacunone	Natural ligand	—	Horiba et al. (2015)
Quinovic acid	Natural ligand	—	Jafri et al. (2016)
Betulinic acid	Natural ligand	1.04	Genet et al. (2010)
Ursolic acid	Natural ligand	1.43	Genet et al. (2010)
Corosolic acid	Natural ligand	0.5	Ladurner et al. (2017)
Maslinic acid	Natural ligand	3.7	Ladurner et al. (2017)
Glycyrrhizic acid	Natural ligand	—	Wang et al. (2017)
Farnesiferol B	Natural ligand	13.53	Kirchweger et al. (2018)
Microlobidene	Natural ligand	13.88	Kirchweger et al. (2018)
RO5527239	Small molecule	0.0036(h)/0.03(m)^a^	Ullmer et al. (2013)
Compound 45h	Small molecule	0.015(h)/0.001(m)^a^	Phillips et al. (2014)
Compound 23g	Small molecule	0.00072(h)/0.0062(m)	Duan et al. (2012)
TRC210258	Small molecule	0.221^a^	Zambad et al. (2013)
Compound 9r	Small molecule	0.00028(h)/0.00092(m)	Zou et al. (2014)
Compound 19	Small molecule	0.034(h)/0.013(m)	Zou et al. (2014)
Compound 18	Small molecule	0.58(h)/0.0247(m)^a^	Briere et al. (2015)
MN6	Small molecule	0.0159(h)/0.0179(m)^a^	Huang et al. (2019)

EC_50_ values (μM) were calculated from at least three independent experiments; h in the table represents the activity of GPBAR1 in human, and m represents mouse; d represents dual agonists.

a Represents EC_50_ value takes the growth of cAMP (a GPBAR1 downstream target) as the GPBAR1 effect indicator.

Apart from specific GPBAR1 agonists, there have been some reports on FXR/GPBAR1 dual agonists, which are also mainly semisynthetic derivatives of BA. We provide a brief summary of several representative dual agonists. The compound INT747, which was first developed in 2002, is a dual ligand of FXR and GPBAR1, but it caused a more intense itch response in clinical trials, so the benefits of this synergistic approach were not realized (Pellicciari et al., 2002). In mice with chronic cholangiopathy, INT-767 can improve liver injury by reducing biliary BA output and promoting HCO_3_^−^ secretion, showing better hepatoprotection than INT-777 (Baghdasaryan et al., 2011). INT-767 activation of FXR induced GPBAR1 gene expression and increased cAMP and Ca^2+^ levels, which then stimulated the secretion of GLP-1, thus improving liver glycolipid metabolism (Pathak et al., 2017). INT-767 demonstrated promising drug-like properties in both liver diseases and metabolic diseases and is considered to be an attractive drug candidate (Rizzo et al., 2010). In a recent study, INT767 was reported to inhibit hepatitis B virus (HBV) infection *in vivo* and *in vitro*, making it a potential anti-HBV candidate (Ito et al., 2021). BAR502 has been shown to reverse steatohepatitis and liver cirrhosis and protect against liver damage induced by a high-fat diet by promoting adipose tissue browning in NASH (Carino et al., 2017). Furthermore, in mice with cholestasis, BAR502 improved survival and reshaped the BA pool without inducing itching (Cipriani et al., 2015). Compared with GPBAR1 agonists alone, the latter two not only showed high efficacy and selectivity in preclinical trials but also showed great application value by significantly avoiding the risk of pruritus in cholestatic models.

### Non-Bile Acid Agonists

The existing GPBAR1 non-BA agonists are mainly developed by ligands, including natural and synthetic ligands (Gertzen et al., 2015; Figure 4; Table 1). Oleanolic acid is one of the rare natural ligands of GPBAR1, showing robust antidiabetic effects on high-fat-fed mice, matching the potential effect of GPBAR1 to improve metabolic disorders (Sato et al., 2007). Ono et al. showed that nomilin (a naturally occurring limonoid) can activate GPBAR1, which is conducive to metabolic regulation (Ono et al., 2011). Similar natural GPBAR1 agonists also include obacunone (Horiba et al., 2015). Quinovic acid is extracted from the national medicine *Fagonia cretica*, and Jafri et al. suggested that all quinovic acid compounds activate the GPBAR1 receptor and further stimulate the secretion of GLP-1 (Jafri et al., 2016). The pentacyclic triterpenoid betulinic acid and ursolic acid are considered selective GPBAR1 agonists (Lo et al., 2016, 2017). Furthermore, Ladurner et al. studied the extracts of 19 kinds of plants, and in addition to oleanolic acid and ursolic acid, their results indicated that corosolic acid and maslinic acid also have activating effects on GPBAR1 (Ladurner et al., 2017). Wang et al. suggested that glycyrrhizic acid extracted from licorice root could activate GPBAR1 and promote the secretion of GLP-1 (Wang et al., 2017). Kirchweger et al. found that two sesquiterpene coumarins, farnesiferol B and microlobidene, can effectively activate GPBAR1, and their activity was equivalent to that of the endogenous ligand lycholic acid, which was considered a potent GPBAR1 agonist (Kirchweger et al., 2018).

Moreover, there have been many reports about small molecule synthesis of GPBAR1 agonists in recent years. Ullmer et al. synthesized an orally bioavailable and selective GPBAR1 agonist, RO5527239, which can effectively improve glucose tolerance and stimulate strong and sustained secretion of GLP-1 (Ullmer et al., 2013). Phillips *et al.* carried out extensive lead optimization from a high-throughput screen hit and ultimately determined that compound 45 h (trifluoromethyl(pyrimidin-2-yl)azetidine-2-carboxamides) was a potent and selective GPBAR1 agonist that plays an effective role in clinical metabolic diseases (Phillips et al., 2014). Similar small molecule agonists also include compound 23 g, TRC210258, compound 9r, compound 19, and compound 18 (Duan et al., 2012; Zambad et al., 2013; Zou et al., 2014; Briere et al., 2015). In recent years, again based on 4-phenoxynicotinamide, researchers synthesized a derivative of 4-phenoxynicotinamide called MN6, which is a potent GPBAR1 agonist. Researchers have suggested that MN6 activates GPBAR1 and further improves insulin sensitivity in skeletal muscles through the cAMP/PKA pathway (Huang et al., 2019). Most of these agonists are based on research achievements in the fields of metabolic disease. How these compounds affect cholestasis liver disease may not be directly known, but current agonist development is still limited to the GPBAR1 exposure range, and more experiments are needed to explore nonsystemic GPBAR1 agonists, which seems to suggest that existing agonists do not have completely specific targets. Of course, in a practical sense, this is the limitation of the status quo of the development of GPBAR1 agonists.

## Discussion and Perspective

BA is an atypical steroid synthesized from cholesterol. Similar to the developmental trajectory of most biochemicals, BA has not been sufficiently recognized in the medical community since the elucidation of the true chemical structure of BA in the 1930s (Hofmann and Hagey, 2014). With the separation and synthesis of DCA and UDCA and the verification of their therapeutic effects on diseases, BA has been gradually used in the treatment of hepatobiliary diseases for nearly half a century. However, with the development of surgical technology, the lack of activity and side effects of UDCA and CDCA greatly reduce the clinical correlation of BA. The discovery of BA-activated receptors seems to have contributed to a BA renaissance (Fiorucci and Distrutti, 2019). FXR and GPBAR1 are two featured BA receptors. To date, there have been many relevant studies on the former and few on the latter. We summarized the roles and pathways of GPBAR1 in cholestatic liver disease.

The BA pool refers to the total amount of BAs circulating in the enterohepatic circulation, including BAs in the liver, gallbladder and intestines, which is a dynamic and complex composition (Rodrigues et al., 2014). Hydrophobic BAs such as CDCA, LCA, DCA and their conjugates have been proved to have high cytotoxicity. The accumulation and distribution of these hydrophobic BAs are closely related to the progress of cholestatic liver disease. Current research shows that GPBAR1 can regulate the size and hydrophobicity of the BA pool. While cholangiocytes are faced with a large amount of hydrophobic BA-induced injury, GPBAR1 can regulate water homeostasis in biliary epithelial cells and restrict the hydrophobicity of the BA pool through cholehepatic shunting. In addition, GPBAR1 promotes the formation of HCO_3_^−^ umbrella and enhances the ability of cholangiocytes to resist hydrophobic BAs. Changes in intercholangiocyte tight junctions (TJs) can lead to leakage of the ducts and bile flow back, causing liver damage. GPBAR1 has also been shown to enhance biliary barrier function by affecting the permeability of TJs. GPBAR1 triggers cell proliferation, which is conducive to maintaining the integrity of the biliary tree under cholestatic liver injury. However, due to the wide expression of GPBAR1 in various cells, GPBAR1 also has potential hazards and can promote tumour cell proliferation, and effective inhibition of GPBAR1 may inhibit tumour cell proliferation and metastasis; however, in cholestatic liver disease, because patients with cholestasis can develop CCA at a later stage, the characteristics of GPBAR1 become intriguing. Moreover, existing research suggests that GPBAR1 can inhibit the progression of cholestasis by inhibiting liver inflammation. The liver is rich in a variety of innate immune cells, including natural killer cells, natural killer T cells, neutrophils, macrophages and DCs (Heymann and Tacke, 2016). The latter three cell types have been confirmed to be involved in cholestatic liver injury and interact with GPBAR1 (Li M. et al., 2017). The NF-κB pathway has long been regarded as a typical proinflammatory signalling pathway that can regulate inflammation through a variety of mechanisms such as the production of proinflammatory factors and leukocyte recruitment, and has been considered the Holy Grail of new anti-inflammatory drugs (Lawrence, 2009). Perhaps for this reason, most studies on the anti-inflammatory pathway of the newly discovered BA membrane receptor GPBAR1 have focused on NF-κB. Inevitably, although the general trend in the findings suggests that GPBAR1 activation can effectively inhibit the development of inflammation, it has also been shown that the activation of GPBAR1 induces the proinflammatory effect of inflammasomes. As the most fully characterized inflammasome thus far, the role of the NLRP3 inflammasome in cholestatic liver disease may require further study. Again, with a flurry of research on the gut microbiome, the relationship between the gut microbiome and cholestatic liver disease has been preliminarily established. GPBAR1 can regulate intestinal metabolism and intestinal inflammation, but the mechanism between GPBAR1 and the gut microbiome in cholestatic liver disease remains to be further validated.

While verifying the function of GPBAR1 is critical, the study of effective GPBAR1 agonists is equally important. In studying GPBAR1 agonists, semisynthetic BA derivatives often manifest stronger GPBAR1 agonist functions than natural BAs, which have been widely used in modern experiments. In addition, extracting natural agonists from plants is also an interesting project. The development of botanical medicine has a long history spanning thousands of years, and through integration with contemporary biotechnology, botanical medicine has been constantly breaking through with pharmacological innovation and has had considerable social impact. Compared with the development of natural GPBAR1 agonists, botanical drugs that act directly on diseases may attract more attention, as traditional Chinese herbs both monotherapies and formulations, have shown favourable efficacy in mouse models of cholestasis (Chen et al., 2015; Ma et al., 2015; Zhao et al., 2015; Yan et al., 2017). Whether Chinese herbal medicine also plays a role in the treatment of cholestasis partly through the activation of GPBAR1 may be an interesting line of inquiry.

Pruritus is one of the main symptoms of cholestasis, but its pathogenesis remains elusive. Some studies have suggested that BA-induced pruritus in cholestasis is associated with GPBAR1 receptor activation, suggesting a potential side effect of GPBAR1 in cholestasis treatment (Lieu et al., 2014; Hussain et al., 2019). But there is evidence that BAs, in addition to activating GPBAR1, require co-activation of multiple receptors and mediators, including TRPA1 channels, to cause pruritus (Lieu et al., 2014). In addition, there have also been studies suggesting that BA-induced pruritus may be linked to activation of FXR. Obeticholic acid is considered the most advanced agonist of FXR and has been approved as a second-line treatment for PBC in 2016. However, it often causes severe pruritus in clinical patients (Fiorucci et al., 2020). Therefore, the current research on the cause of pruritus caused by BA is still controversial in many aspects. We may not be able to roughly define the complex relationship between GPBAR1 and pruritus. It is gratifying that the combination of BA agonists with other drugs and the use of BA receptor dual agonists seems to significantly reduce the pruritus in cholestasis. In addition to effectively controlling the pruritus induced by BA, some GPBAR1/FXR dual agonists limits the nonselective proliferation of GPBAR1 since they limits the overexpression of GPBAR1. Both FXR and GPBAR1 are BA-activated receptors that have attracted wide attention. Activation of these receptors leads to different pathway regulation, double activation combines the functions of individual activation, and adaptive generation does not play an additional role in individual activation. These dual agonists tend to show better therapeutic efficacy than single agonists and induce fewer side effects (Wang et al., 2018). Therefore, dual targeting of the BA membrane receptor GPBAR1 and nuclear receptor FXR may be a promising strategy for cholestatic liver disease.

In conclusion, GPBAR1 can protect the liver from the effects of BA overload and effectively control liver inflammation. Then, GPBAR1 can stimulate cell regeneration to maintain the integrity of cholangiocytes and regulate the hydrophobicity of the BA pool. Additionally, GPBAR1 is involved in regulating gut metabolism and inflammation. GPBAR1 appears to form a signalling network between the liver, gallbladder and gut to alleviate cholestatic liver injury.

## Glossary

ABAT: apical BA transporter
AE2: anion exchanger 2
ALP: alkaline phosphatase
AQP2: aquaporin-2
ASBT: apical sodium-dependent BA transporter
BA: bile acid
BDL: bile duct ligation
CA: cholic acid
CCA: cholangiocarcinoma
CDCA: chenodeoxycholic acid
CFTR: cystic fibrosis transmembrane conductance regulator
DCA: deoxycholic acid
DCs: dendritic cells
ECC: extrahepatic cholangiocarcinoma
EGFR: epidermal growth factor receptor
eNOS: endothelial nitric oxide synthase
EPACs: exchange proteins activated by cAMP
GCA: glycocholic acid
GCDCA: glycochenodeoxycholic acid
GDCA: glycodeoxycholic acid
GGT: gamma-glutamyl transferase
GLCA: glycolithocholic acid
GLP1: glucagon-like peptide-1
GPCRs: G protein-coupled receptors
GSK3β: glycogen synthase kinase 3β
InsP3R: inositol 1,4,5-trisphosphate receptor
iNOS: inducible nitric oxide synthase
KO: knockout
LCA: lithocholic acid
LPS: lipopolysaccharide
MRP2: multidrug resistance-associated protein 2
MRP4: multidrug resistance-associated protein 4
NASH: non-alcoholic steatohepatitis
NO: nitric oxide
PBC: primary biliary cirrhosis
PH: partial hepatectomy
PKA: protein kinase A
PSC: primary sclerosing cholangitis
ROS: reactive oxygen species
TCA: taurocholic acid
TCDCA: taurochenodeoxycholic acid
TDCA: taurodeoxycholic acid
TJPs: tight junction proteins
TLCA: taurolithocholic acid
UDCA: ursodeoxycholic acid
WT: wild-type
